# Project Extension for Community Health Outcomes (ECHO) Autism: A Successful Model to Increase Capacity in Community-Based Care

**DOI:** 10.3390/brainsci12030327

**Published:** 2022-02-28

**Authors:** Kristin Sohl, Agnieszka Rynkiewicz, Valeria Nanclares-Nogués, Alicia Brewer Curran, Julie Scorah, Mandy Steiman, Catherine Lord, Roma A. Vasa, Agnieszka Słopień, Małgorzata Janas-Kozik, Izabela Łucka, Artur Mazur

**Affiliations:** 1 ECHO Autism Communities, Department of Child Health, University of Missouri School of Medicine, Columbia, MI 65211, USA; sohlk@health.missouri.edu (K.S.); vnbhp@missouri.edu (V.N.-N.); currana@health.missouri.edu (A.B.C.); 2 Department of Psychiatry, College of Medical Sciences, University of Rzeszow, 35-956 Rzeszow, Poland; 3 Center for Diagnosis, Therapy and Education SPECTRUM ASC-MED, 80-404 Gdansk, Poland; 4 Montreal Neurological Institute-Hospital, Azrieli Centre for Autism Research McGill University, Montreal, QC H3A 2B4, Canada; julie.scorah@muhc.mcgill.ca (J.S.); mandy.steiman@muhc.mcgill.ca (M.S.); 5 Semel Institute for Neuroscience & Human Behavior, University of California Los Angeles, Los Angeles, CA 90095, USA; clord@mednet.ucla.edu; 6 Center for Autism and Related Disorders, Kennedy Krieger Institute, John Hopkins University School of Medicine, Baltimore, MD 21205, USA; vasa@kennedykrieger.org; 7 Department of Child and Adolescent Psychiatry, Poznan University of Medical Sciences, 61-701 Poznan, Poland; agaslopien@ump.edu.pl; 8 Department of Psychiatry and Psychotherapy, Medical University of Silesia, 40-635 Katowice, Poland; janaskozik@gmail.com; 9 John Paul II Paediatric Centre, 41-218 Sosnowiec, Poland; 10 Department of Developmental Psychiatry, Psychotic and Geriatric Disorders, Medical University of Gdansk, 80-282 Gdansk, Poland; izabelalucka@wp.pl; 11 Department of Paediatrics, Paediatric Endocrinology and Diabetes, College of Medical Sciences, University of Rzeszow, 35-959 Rzeszow, Poland; cm@ur.edu.pl

**Keywords:** autism spectrum disorder, ECHO Autism, health care access, screening, global programs, primary care providers, cross-disciplinary care

## Abstract

Individuals with autism spectrum disorder (ASD) struggle to access high-quality health care due to the shortage of trained providers. ECHO (*Extension for Community Healthcare Outcomes)* Autism is a unique educational program that allows ASD experts to provide knowledge and skills to professionals in local communities to deliver evidence-based care to children with ASD and their families. The model teaches clinicians how to screen and diagnose ASD, as well as manage common co-occurring medical and mental health issues. ECHO Autism is particularly useful for addressing the complex needs of children with ASD and reducing disparities often present in rural and underserved communities. The model can be disseminated globally due to its flexibility in accommodating local and regional differences in social norms and constructs. This article provides an overview of the format of the ECHO Autism model, data supporting the model’s efficacy, and discusses future research directions.

## 1. Introduction

Autism Spectrum Disorder (ASD) is a neurodevelopmental disorder associated with significant impairments in social, communication, and behavioral functioning [1]. ASD requires comprehensive care, often from multiple healthcare providers, as it is associated with medical and psychiatric comorbidities at a higher rate than other neurodevelopmental challenges such as depressive and anxiety disorders, sleep dysfunction, seizures, ED, attention-deficit/hyperactivity disorder, tic disorders, among others [2,3]. ASD is a lifelong condition associated with higher morbidity [4]. According to the latest estimates, for example, autistic females are several times more likely to die by suicide than non-autistic females [5]. The global prevalence estimate of ASD is 1–2% [6] and the Centers for Disease Control (CDC) now estimates that 1 in 44 children in the US are identified with ASD, with an average age of diagnosis of just over four years of age [7].

The rising prevalence over the last couple of decades [8] has led to an increased demand for evaluations that has outpaced the number of specialists and specialty centers, often leading to significant waitlists and barriers for families [9,10,11]. Long wait times for services is particularly problematic given that early intervention has been demonstrated to improve outcomes in children with ASD [12,13]. Therefore, given the complexity of ASD and the fact that there are insufficient numbers of specialists to meet the demand, individuals with ASD and their families continue to be at high risk for unmet healthcare needs. This is especially true in rural or less developed areas of the US, but even more pronounced throughout developing countries across the world. Healthcare barriers include scarcity of resources (lack of trained diagnostic and intervention clinicians), higher cost of services, and often cultural or language barriers leading to delays in access to care [14]. In addition, many families must travel great distances to obtain the necessary diagnostic and intervention services, further impacting access to the right care at the right time [15].

The ECHO Autism program has reimagined the system of care by increasing access to high-quality healthcare and education. ECHO Autism programs equip community-based professionals, such as physicians, nurse practitioners, psychologists, allied health professionals, educators, and advocates, with the knowledge and confidence to provide autistic people and their families across the globe best-practice care, education, and advocacy, with the ultimate goal of empowering people with autism and their families to live their best lives.

## 2. Overview of the ECHO Model^®^

Project ECHO^®^ (Extension for Community Healthcare Outcomes) was founded by Dr. Sanjeev Arora, a liver specialist at the University of New Mexico, in 2003. Arora developed the model in response to the needs of patients with hepatitis C virus who lived in remote and underserved areas, as a way of bringing best care practices to the patient, rather than having the patient travel to specialists [16]. The ECHO model is a mentorship model that democratizes specialists’ knowledge and empowers generalists to provide expert care to local communities in the areas of healthcare, education, and public service. The model’s design is a “hub and spoke” framework, where an interdisciplinary “hub team” of content experts provides guided practice to “spokes”, or professionals in local communities, to build capacity. TeleECHO sessions are held regularly via videoconferencing technology. Core features of a teleECHO session include a brief didactic, typically presented by a content expert on the hub team, and deidentified case-based presentations delivered by spokes, where rich, interactive discussions occur from hub team members and peer spokes alike. The ECHO model is not telemedicine, where one-to-one care is delivered; rather, it is a telementoring model, where specialists provide guided practice to generalists. The ECHO model has now been replicated across the United States and countries around the world, covering over 100 complex conditions, such as HIV, COVID-19, diabetes, chronic pain, endocrinology, addiction, and behavioral health disorders and psychiatric conditions, with over 400 replication sites globally. Though the ECHO model was originally designed for healthcare, implementation is scaling to meet the demands of a diverse, global population with scarce access to a multitude of resources. Project ECHO is committed to improving equitable access to best practices to bridge the research to clinical practice gaps and allow more individuals to experience the right care at the right time. Project ECHO’s funding reflects its global conceptualization of serving the underserved. Therefore, programs are not permitted to charge or profit from Project ECHO programs. Project ECHO programs work across a diverse resource network including local and centralized governments, philanthropy and granting agencies to achieve improved outcomes.

## 3. ECHO Autism Framework

Given the increasing prevalence of ASD and the resultant impact on identification and intervention services, it is critical to develop innovative models that can meet the current demand. When applied to autism, the ECHO model is particularly useful for addressing the complex needs of children with ASD and reducing disparities often present in rural and underserved communities [7,17,18]. In 2015, the original ECHO Autism pilot program [17] was developed by the University of Missouri to increase general practitioners’ self-efficacy in screening and identifying ASD, as well as screening and managing common co-occurring conditions in individuals with ASD. Results showed significant increases in percentage of pediatrician compliance with established general developmental and autism screening guidelines, increasing from 30% compliance to 60% compliance in a 6-month period. In addition, the results demonstrated that the ECHO Autism framework is both feasible and effective for increasing capacity for best-practice medical care for children with ASD [17]. Further, general practitioners (GPs) reported a decreased number of perceived barriers to caring for children with autism in their practice. These findings are particularly important when considering programs that can guide GP practice change to increase access and reduce disparities for the underserved.

In an effort to create efficient access to high-quality diagnosis, the ECHO Autism program created a structure for GPs who desired to further develop their expertise and provide evaluation and diagnosis to children with a suspicion of ASD. The ECHO Autism: STAT (Screening Tool for Autism in Toddlers and Young Children) [19] program targets children 14 to 48 months with unambiguous symptoms of autism. Results demonstrated a decrease in barriers associated with prompt diagnosis and an increase in access to services, an average of 2 to 6 months sooner than if children had received an evaluation at an autism center in the region [18]. An additional key benefit was the reduction in the burden of travel for families by an average of 173 miles [18], reflecting the notable reach of this model in increasing access to care in rural or underserved communities. Additionally, ECHO Autism: STAT GPs demonstrated better adherence to the American Academy of Pediatrics (AAP) general developmental and autism screening guidelines as compared to the participants in the pilot study, as they achieved a 95% adherence rate to AAP screening guidelines at 12 months [20]. This model is about equipping the GPs to care for ASD patients through best practices, regardless of geographic location. Similarly, the College of Medical Sciences, University of Rzeszow, the leader of ECHO Autism in Poland provides translated STAT material [21,22] for free to all the replication sites in Poland. ECHO Autism will accelerate the work carried out in Poland by continuing to provide professional development and case-based discussions between specialists and generalists as they deliver autism best practices.

One of the keystones of the ECHO Autism program is patient- and family-centered care. To model the importance of shared decision making and to demonstrate the delivery of patient- and family-centered care, the University of Missouri ECHO Autism Primary Care program was the first to include a parent of a person with ASD and an autistic person as a content expert on the hub team. These voices were so highly valued by peer hub team members and participants that it became standard practice for all ECHO Autism programs to include at least one person with lived experience on the hub team. Though the addition of this unique content expert was an adaptation from the original ECHO model, ECHO programs outside of ASD have followed the standard set by ECHO Autism to include a person with lived experience due to the richness they add to the learning of participants through case discussions and brief didactics they present. ECHO Autism is proud to have paved the way for the patient and family voice to be regarded so highly and become a trend across the meta-ECHO Community.

## 4. Global Scale of ECHO Autism

The ECHO Autism framework has now been replicated across the United States (US) and around the world. In 2016, ECHO Autism, in collaboration with the Autism Speaks Autism Treatment Network (AS-ATN) and the Autism Intervention Research Network on Physical Health (AIR-P), launched a large-scale prospective multisite study across ten replication trial sites in the US and Canada. Although results from the 6-month intervention trial did not demonstrate statistically significant screening practice pattern changes as measured by participating clinician chart review, the ECHO Autism model demonstrated a reduction in clinician perceived barriers to providing care to children with ASD, as well as marked improvements in clinician knowledge and confidence in treating children with ASD [23].

The replication trial served as a springboard for global expansion of ECHO Autism programs. The University of Missouri ECHO Autism Communities team is currently a global Superhub (authorized ECHO training and technical assistance hub) for autism- and disability-focused ECHO programs. The flexibility of the model allows for cultural adaptation related to autism best practices and local or regional differences in social norms and constructs. As professionals across disciplines and locations receive guided practice through ECHO Autism programs, they extend their service into local communities. To date, the University of Missouri team leads the ECHO Autism Collaborative. The Collaborative comprises over 60 organizations who are developing an ECHO Autism program or have active programs established. Approximately a third of those organizations are based outside of the United States. Global ECHO Autism programs support community professionals who work with people who have ASD across the lifespan. Examples of ECHO Autism program offerings include Primary Care, Psychology, Early Intervention, Education, Extremism, Behavior Solutions in Schools, Employment, Research Units in Behavioral Intervention (RUBI), Crisis Care, Mental Health, Applied Behavior Analysis, Transition to Adulthood, Adult Healthcare, Caregiver Skills Training, Family Advocates, and Center Engagement. The synergy between the number of programs is laying a foundation for ECHO Autism Communities program networks, where professionals coordinate care to best support autistic people and their families across the lifespan [24,25,26].

## 5. Conclusions and Future Directions

The ECHO Autism framework is showing promise in reducing barriers and disparities by increasing the capacity of the local autism workforce through training and skill development. The ECHO Autism framework has been highly regarded across international replication sites, with high attendance rates and low attrition. ECHO Autism programs in Argentina, Chile, India, Uruguay and Kenya engaged participants across their geographic regions to accelerate dissemination of autism best practices. We are encouraged by the positive impact that the model has on professional development, both in terms of increased self-efficacy with autism best-practices and in promoting cross-disciplinary care. As more generalists engage with specialists through ECHO Autism, we anticipate increasing ECHO Autism Communities networks to support access to best practices at the patient level. Many ECHO Autism programs are designed for identification of ASD and appropriate follow-up care in young children. Given the rising adult ASD population, it is particularly important to create more programs for adolescent and adult populations such as those focused on management of comorbidities and the transition to adulthood.

A known limitation of Project ECHO research is the absence of Patient-Reported Outcomes (PROs). The ECHO Autism framework has strong evidence supporting ECHO Autism programs as successful in improving generalist clinician self-efficacy, knowledge, and reduced perceived barriers to caring for children with ASD. ECHO Autism research has not yet focused on the outcomes of those served by ECHO Autism clinicians and professionals. However, by increasing clinician knowledge in best practice care and decreasing barriers for both families and clinicians, it is expected that PROs will also support the effectiveness of the model, delivering the right care, at the right time, in their own community. Research focused in this area is a future priority of ECHO Autism Communities research. Additionally, the ECHO Autism partners around the world are working on developing randomized control trials (RCTs) to evaluate the effectiveness of these programs to improve functionality and clinical outcomes in autism spectrum disorders.

## Data Availability

Not applicable.
